# Is Combined Tranexamic Acid Administration Superior to Single-Route Protocols in Primary Total Knee Arthroplasty? A Prospective Cohort Study

**DOI:** 10.3390/jcm15124776

**Published:** 2026-06-19

**Authors:** Zeljko Stepanovic, Branko Ristic, Aleksandar Matic, Nikola Prodanovic, Jelena Milosevic, Ivan Stojadinovic, Nikola Andric, Tijana Prodanovic, Bojan Milenkovic, Dragan Knezevic, Djordje M. Kolak

**Affiliations:** 1 Department of Surgery, Faculty of Medical Sciences, University of Kragujevac, 69 Svetozara Markovića St., 34000 Kragujevac, Serbia; stepakg@gmail.com (Z.S.); branko.ristic@gmail.com (B.R.); maticaleksandar@gmail.com (A.M.); ivan_stojadinovic@yahoo.com (I.S.); andricnikola91kg@gmail.com (N.A.); dragankg984@gmail.com (D.K.); djordje.kolak@gmail.com (D.M.K.); 2 University Clinical Center Kragujevac, 30 Zmaj Jovina St., 34000 Kragujevac, Serbia; jecas0109@gmail.com (J.M.); tijanaprodanovic86@gmail.com (T.P.); 3 Department of Physical Medicine, Faculty of Medical Sciences, University of Kragujevac, 69 Svetozara Markovića St., 34000 Kragujevac, Serbia; 4 Department of Pediatrics, Faculty of Medical Sciences, University of Kragujevac, 69 Svetozara Markovića St., 34000 Kragujevac, Serbia; 5 Department of Orthopedics and Traumatology, University Hospital Medical Center Bežanijska Kosa, Bežanijska Kosta St., 11080 Belgrade, Serbia; bojanmilenkovic89@gmail.com

**Keywords:** total knee arthroplasty, tranexamic acid, hidden blood loss, administration route, multivariate analysis, blood management, perioperative bleeding

## Abstract

**Background**: The optimal route of tranexamic acid (TXA) administration remains one of the most debated topics in total knee arthroplasty. This study aimed to compare the effects of intravenous (IV), intra-articular (IA), and combined TXA protocols on total blood loss (TBL) and hidden blood loss (HBL), while identifying independent predictors of perioperative bleeding. **Methods**: In a prospective cohort study of 245 patients undergoing primary TKA, participants were assigned into four groups: IV TXA (15 mg/kg), IA TXA (1 g), combined (IV + IA), and a control group. TBL and HBL were calculated using the Gross formula. A multivariate linear regression model was used to assess independent associations of each protocol. **Results**: The IV group demonstrated significantly lower TBL (mean 898 mL) and HBL (mean 568 mL) compared with both the control (1329 mL and 894 mL; *p* = 0.002) and IA groups (1129 mL and 748 mL; *p* = 0.008). While IA TXA reduced 24 h drain output (*p* < 0.001), it did not significantly reduce TBL (*p* = 0.539) or HBL (*p* = 0.875). No significant differences were found between the IV-only and combined groups (*p* > 0.05). Multivariate regression identified the IV route as an independent predictor of reduced TBL (B = −383.7, *p* = 0.001). **Conclusions**: A single intravenous dose of TXA was associated with lower total and hidden blood loss compared with intra-articular administration. The lack of additional benefit in the combined group suggests a possible plateau effect of systemic administration, which is hypothesis-generating and limited by the study design for blood conservation in TKA. **Level of Evidence**: Level II, Prospective Cohort Study.

## 1. Introduction

Total knee arthroplasty (TKA) is considered the most effective treatment for severe knee osteoarthritis (OA), with long-term results rated as excellent or good in over 98% of cases [1,2]. While there have been significant improvements in surgical techniques, the amount of blood lost during TKA remains a major concern for surgeons [3,4]. Sehat et al. introduced the concept of hidden blood loss (HBL) that accounts for approximately 50% of the total calculated blood loss in TKA procedures [5,6]. Increased HBL is associated with more severe pain, higher risks of hematoma formation, wound infections, and diminished knee joint mobility [7]. On average, the total blood loss can reach up to 1500 mL, which contributes to a high rate of blood transfusions [8,9,10]. More than 70% of patients who undergo joint replacement surgery receive a blood transfusion [11,12,13]. Receiving allogenic blood can increase the risk of longer hospital stays, transmitting infectious diseases, immune reactions, hemolytic responses, anaphylactic reactions, and even higher mortality rates [10].

Tranexamic acid (TXA), a synthetic antifibrinolytic agent, has been shown to effectively reduce blood loss and transfusion requirements in TKA. Both intravenous (IV) and topical intra-articular (IA) administration routes are widely used and supported by the literature [9,14]. Intravenous TXA provides systemic inhibition of fibrinolysis, whereas intra-articular application targets local fibrinolytic activity within the surgical field [15]. Although both modalities have demonstrated efficacy and safety, there is still no clear consensus regarding the optimal route of administration.

A separate randomized study demonstrated that topical TXA was similar in efficacy to intravenous TXA for controlling blood loss [16]. Another randomized controlled trial demonstrated that combined intra-articular and intravenous TXA reduced blood loss in total knee arthroplasty, with no thromboembolic complications [17,18]. Alshryda et al. performed a meta-analysis of randomized controlled trials and found that intravenous infusion of TXA during TKA does not support an increased risk of deep-vein thrombosis or pulmonary embolism due to TXA administration [19].

Several studies and meta-analyses have suggested that combined IV and IA administration of TXA may provide superior blood-sparing effects compared with a single route [17,18,19,20]. However, the available evidence is limited by heterogeneous study designs, variable dosing regimens, and the frequent absence of a placebo control group [21].

In addition, there is considerable variability in clinical practice regarding TXA dosing regimens and routes of administration, which reflects the absence of universally accepted protocols in primary TKA. Therefore, further comparative clinical evidence is needed to optimize perioperative blood management strategies in routine arthroplasty practice.

This prospective comparative cohort study aimed to compare intra-articular, intravenous, and combined administration of tranexamic acid with placebo on perioperative blood loss in primary TKA. We hypothesized that combining IV and IA TXA administration would be superior in reducing total blood loss and postoperative hemoglobin decrease compared with single-route administration and placebo, without increasing thromboembolic complications.

## 2. Materials and Methods

### 2.1. Study Design and Study Group

This prospective, single-center cohort study was conducted at the University Clinical Center Kragujevac, Serbia. Institutional Ethics Committee approval was obtained on 8 December 2025 (No. 01/25-836), prior to the initiation of patient recruitment, and all patients provided written informed consent. The study was designed as a prospective observational cohort study reflecting real-world clinical practice, without randomization or experimental allocation of treatment. The study followed the Declaration of Helsinki and was reported in accordance with STROBE guidelines.

A priori power analysis determined the required sample size to detect a clinically relevant 200 mL difference in hidden blood loss (HBL) between groups, based on the previous literature [22]. Assuming a standard deviation of 200 mL, α = 0.05, and power of 0.80, one-way ANOVA indicated a minimum of 180 patients (45 per group). Allowing 15% attrition, target enrolment was set at 212 patients.

Consecutive patients scheduled for primary unilateral TKA for end-stage osteoarthritis were screened. Osteoarthritis was defined clinically and radiographically as Kellgren–Lawrence grade III or IV [23]. Inclusion criteria were age ≥ 40 years and indication for primary unilateral TKA. Exclusion criteria included rheumatoid arthritis, severe deformity, revision or bilateral TKA, allergy to tranexamic acid (TXA), thromboembolic disease within one-year, active malignancy, severe renal insufficiency, coagulopathy, or anticoagulation therapy that could not be discontinued perioperatively.

**Figure 1:**
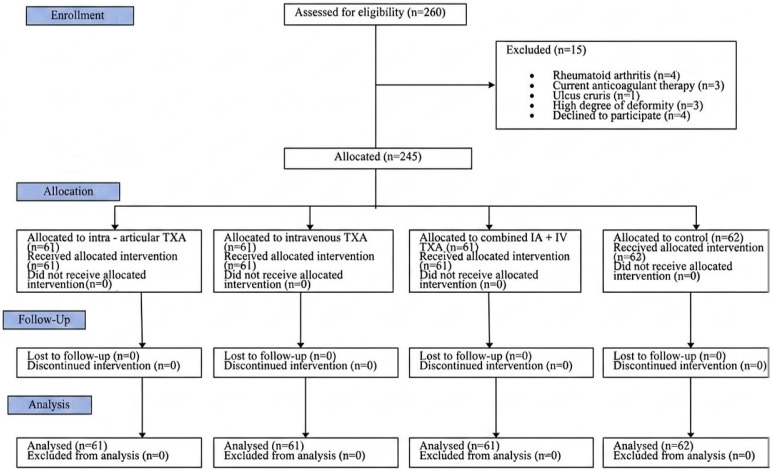
Patient recruitment and allocation flow chart.

Between 9 December 2025 and 15 April 2026, 260 patients were assessed; 245 met the inclusion criteria (Figure 1). Patients were assigned to four groups: intra-articular TXA (IA; *n* = 61), intravenous TXA (IV; *n* = 61), combined IA + IV TXA (*n* = 61), and control (*n* = 62). Group allocation was performed according to predefined institutional perioperative anesthesia–orthopedic protocols, without randomization or patient-specific selection criteria. The decision regarding the administration route was made by the attending orthopedic surgeon and anesthesiologist based on an institutional perioperative TXA protocol and routine clinical assessment. Allocation to intravenous, intra-articular, combined IV + IA, or control groups reflected predefined institutional treatment pathways, where IV administration was used as the standard systemic protocol, IA administration as a local hemostatic strategy, combined administration in cases where enhanced blood conservation was clinically preferred, and the control group included patients who did not receive TXA as part of routine care. The decision process was not influenced by study participation. The intra-articular tranexamic acid dose of 1 g was selected based on previously published clinical evidence and commonly used dosing regimens in total knee arthroplasty demonstrating efficacy and safety [24]. The final sample provided post hoc power exceeding 85% for the detection of between-group differences.

### 2.2. Surgical Technique and Rehabilitation

All patients underwent primary unilateral TKA using a standardized medial parapatellar approach. Limb exsanguination was performed using a sterile elastic tourniquet (HemaClear^®^, OHK Medical Devices, Haifa, Israel) prior to incision and released before wound closure. The same implant design (NexGen^®^ Legacy Posterior Stabilized (LPS) system, Zimmer Biomet, Warsaw, IN, USA) and cementing technique were used in all cases. The wound was closed in a standardized manner after meticulous hemostasis. A closed suction drain was applied in all patients and was managed according to a uniform postoperative protocol across all study groups.

Perioperative regional anesthesia and postoperative rehabilitation protocols were identical across groups. Isometric exercises were initiated shortly after the operation. The suction drain was removed on the first postoperative day, followed by the initiation of active and assisted range-of-motion exercises. Full weight-bearing ambulation started on the first postoperative day.

Blinding was not feasible due to the nature of the intervention. Postoperative transfusion decisions were based on a predefined institutional protocol (hemoglobin < 80 g/L or symptomatic anemia), ensuring standardized postoperative management across all study groups.

**Figure 2:**
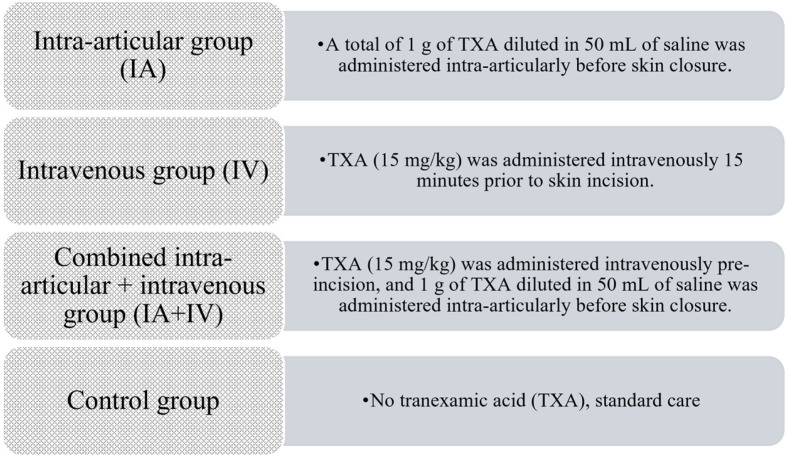
Tranexamic acid administration protocols.

A schematic diagram of the drug administration protocol was developed according to groups, providing a clear overview of the therapeutic regimens. The figure includes the basic elements of drug administration, including dosage and route of administration (Figure 2).

### 2.3. Perioperative Protocol

Standardized prophylaxis included enoxaparin (4000 IU SC daily) starting 12 h postoperatively, and antibiotic prophylaxis with cefazolin (1 g IV every 8 h for 24 h) or vancomycin for allergic patients. Postoperative analgesia consisted of scheduled IV paracetamol (1000 mg every 8 h) and rescue tramadol (100 mg every 8 h) for breakthrough pain. In the IA, combined (IV + IA), and control groups, wound drains were clamped for 3 h postoperatively before being released as part of a uniform institutional postoperative protocol applied across all study groups to standardize early postoperative drainage conditions. Patients in the control group did not receive tranexamic acid and underwent the same standardized perioperative management protocol as the active treatment groups.

### 2.4. Assessment and Transfusion

Hemoglobin levels were recorded preoperatively and 24 h postoperatively. Blood transfusion was performed according to the institutional threshold (Hb < 8 g/dL or symptomatic anemia) and based on standardized, predefined transfusion criteria applied uniformly across all study groups. All patients were followed for 6 weeks to monitor adverse effects, local complications, and signs of venous thromboembolism (VTE). Outcome assessment was performed using routine clinical follow-up and standardized hospital records.

### 2.5. Outcome Measures

Clinical data included demographic characteristics, preoperative status, and postoperative outcomes up to six weeks. Primary outcomes were drainage volume, total blood loss (TBL), hidden blood loss (HBL), intraoperative blood loss, and allogenic transfusion rate. TBL was calculated using Gross’s equation based on the difference between preoperative and postoperative hematocrit [25]:

TBL = EBV × (Hct_pre − Hct_post)/Hct_ave.

Estimated blood volume (EBV) was calculated using Nadler’s formula for men and women based on height and weight [22]. Hidden blood loss was calculated according to Sehat et al. [5]:

HBL = TBL − (intraoperative blood loss + drainage volume).

Secondary outcomes included postoperative hemoglobin drop, transfusion requirement, thromboembolic events, and wound complications. Hemoglobin drop was defined as the difference between preoperative and first postoperative day values and was recorded using standardized laboratory measurements performed in the institutional clinical laboratory.

### 2.6. Statistical Analysis

Statistical analyses were performed using SPSS (Version 26.0, IBM Corp., Armonk, NY, USA) while the R program was used to create the forest plot. Data are presented as numbers and percentages for categorical variables, or as median and interquartile range (IQR) for numerical variables. The normality of the distribution was tested with the Kolmogorov–Smirnov test. The Pearson χ^2^ test or Fisher’s exact test was used to compare categorical variables between groups, while the Kruskal–Wallis test was used to compare numerical variables between more than two groups. Subsequent comparisons between groups were performed by the Mann–Whitney U test with Bonferroni correction for multiple comparisons. Spearman correlation was used to assess the association between numerical variables. A multivariate linear regression analysis was conducted in order to examine the independent association of the examined factors with total blood loss and was adjusted for baseline demographic and perioperative covariates. Statistical significance was defined at the level of *p* < 0.05 and all tests were two-sided.

## 3. Results

### 3.1. Patient Characteristics

A total of 245 patients were included in the study, of which 146 (59.6%) were female, and 99 (40.4%) were male. Baseline demographic and clinical characteristics were comparable across all study groups. Patient characteristics, overall and by group, are shown in Table 1.

### 3.2. Hematological Outcomes

Laboratory parameters of patients in relation to the method of TXA administration are shown in Table 2.

### 3.3. Blood Loss Outcomes

**Figure 3:**
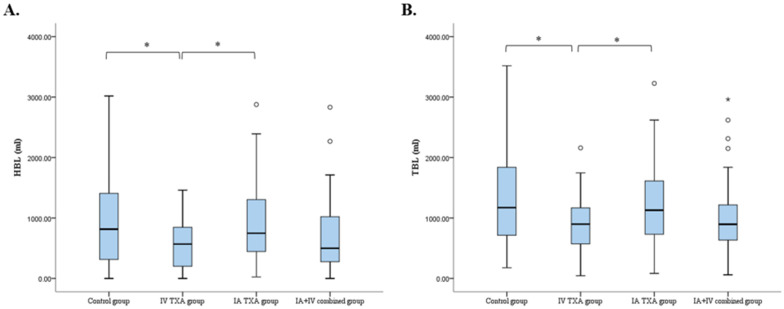
Distribution of blood loss according to the method of administration of tranexamic acid. (**A**) Hidden blood loss (HBL). (**B**) Total blood loss (TBL). Statistical significance is indicated as * *p* < 0.05.

Statistically significant differences were observed across all primary blood loss outcomes, including total blood loss (TBL), hidden blood loss (HBL), and 24 h drainage volume (Kruskal–Wallis, *p* < 0.05; Table 3). Post hoc analysis showed that the IV TXA group had significantly lower TBL (*p* = 0.002) and HBL (*p* = 0.019) compared with the control group. In addition, IV administration resulted in lower TBL (*p* = 0.009) and HBL (*p* = 0.008) compared with the IA group (Figure 3). The IA group demonstrated significantly reduced drain output versus controls (*p* < 0.001), but no significant effect on TBL (*p* = 0.539) or HBL (*p* = 0.875). No significant differences were observed between IV and combined (IV + IA) administration for TBL (*p* = 0.553) or HBL (*p* = 0.619), indicating no additional benefit of combined dosing. Parameters of blood loss and drainage in relation to the method of TXA administration are shown in Table 3.

Distribution of blood loss according to the method of administration of tranexamic acid is shown in Figure 3.

Boxes represent the interquartile range, horizontal lines the median, and whiskers the range of values.

**Figure 4:**
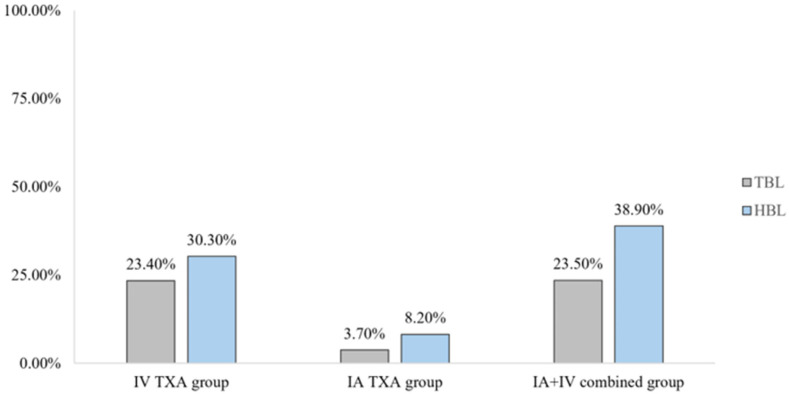
Percentage reduction in total blood loss (TBL) and hidden blood loss (HBL) in IV, IA, and combined tranexamic acid groups compared with the control group.

Figure 4 shows percentage reduction in TBL and HBL compared with the control group.

**Figure 5:**
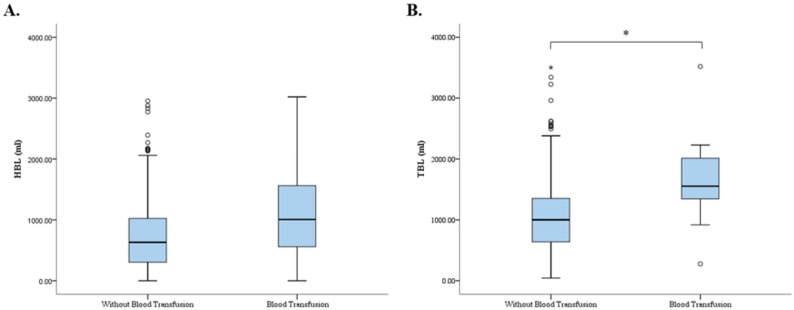
Distribution of blood loss in relation to the use of blood transfusion. (**A**) Hidden blood loss (HBL). (**B**) Total blood loss (TBL). Statistical significance is indicated as * *p* < 0.05.

Patients who received a blood transfusion had significantly higher total blood loss (TBL) values compared to non-transfused patients. Median TBL was 1554 mL (IQR 989.0) in transfused patients versus 1001 mL (IQR 727.5) in non-transfused patients (Mann–Whitney, *p* = 0.023). Regarding hidden blood loss (HBL), no statistically significant difference was shown (Mann–Whitney, *p* = 0.212). Distribution of blood loss in relation to the use of blood transfusion. (A) Hidden blood loss (HBL). (B) Total blood loss (TBL) is shown in Figure 5.

**Figure 6:**
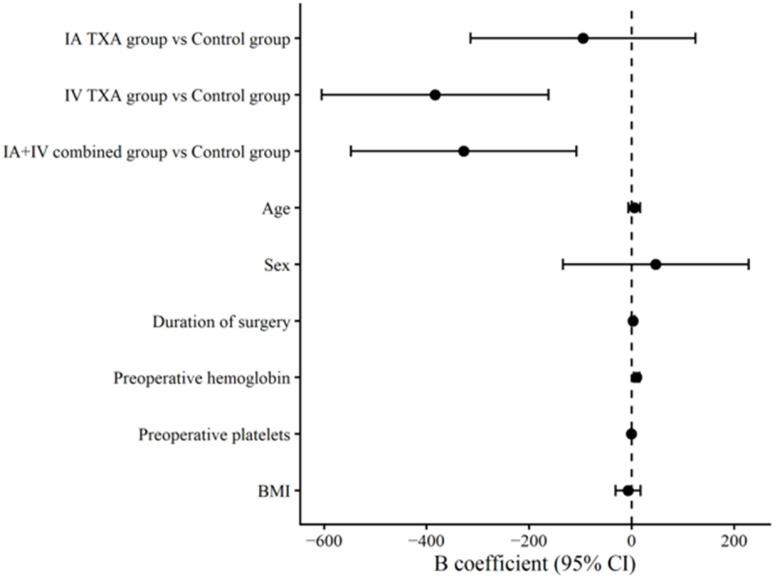
Forest plot of multivariate linear regression analysis for TBL.

The following variables were included in a multivariate linear regression model with TBL as the dependent variable: treatment group (intra-articular TXA vs. control, intravenous TXA vs. control, and combined TXA vs. control), age, sex, duration of surgery, preoperative hemoglobin, preoperative platelets, and BMI. The model was statistically significant (F = 4.533; *p* < 0.001) and explained 14.8% of the variability in total blood loss (R^2^ = 0.148; corrected R^2^ = 0.115). Compared to the control group, intravenous TXA was associated with an average of 383.8 mL lower TBL, while combined TXA administration was associated with an average of 327.8 mL lower TBL, controlling for age, gender, BMI, duration of surgery, preoperative hemoglobin, and platelets. Multivariate linear regression analysis of factors associated with total blood loss (TBL) is shown in Table 4 and Figure 6.

Multivariate linear regression analysis was performed to identify independent predictors of total blood loss. After adjusting for age, gender, BMI, and operative time, only the administration of IV tranexamic acid (B = −383.7, *p* = 0.001) and combined TXA (B = −327.8, *p* = 0.004) remained significant independent factors for blood loss reduction. Intra-articular administration alone did not significantly predict blood loss outcomes (*p* = 0.394). Additionally, higher preoperative hemoglobin levels were independently associated with increased calculated total blood loss (B = 9.5, *p* = 0.001). Points represent the B coefficients, and horizontal lines represent the 95% confidence intervals (CIs). Systemic TXA administration (IV and combined) demonstrates a significant independent reduction in blood loss, as their confidence intervals do not cross the null line.

Forest plot of the multivariate linear regression analysis for predictors of total blood loss is shown in Figure 6.

### 3.4. Complications

Patients were followed up for 6 weeks postoperatively. There were no patients with symptoms of thromboembolism or wound infection. Complications related to bleeding, including wound discharge, secretion of drain removal site, and hematoma did not occur. There were no adverse events associated with TXA such as pale skin, unusual tiredness or weakness, nausea, vomiting, or hypersensitivity in any of the patients.

## 4. Discussion

Our results suggest that intravenous (IV) administration of tranexamic acid (TXA) is associated with a greater reduction in total blood loss and hemoglobin drop compared to both intra-articular and control groups, while showing no statistically significant difference from the combined TXA protocol. These findings suggest that a single-dose IV TXA may represent an effective approach for blood conservation in total knee arthroplasty, as the addition of a local dose did not demonstrate a statistically significant additional benefit.

### 4.1. Patient Characteristics

Baseline demographic and clinical variables were comparable across groups and were consistent with previously published TKA cohorts [7,20,26]. This indicates that baseline characteristics were balanced across groups, reducing the likelihood of confounding in the observed blood loss outcomes.

### 4.2. Hematological Outcomes

The observed fluctuations in postoperative hemoglobin (Hb) and hematocrit reflect the expected perioperative changes associated with blood loss estimation. While preoperative baselines were uniform, the attenuated Hb decline in the IV TXA group and the combined TXA group (22–23 g/L vs. 28 g/L in controls) was observed as a numerically lower decline compared with controls. The unchanged hematocrit drop in the IA and control groups supports the limited systemic impact of local TXA, consistent with previous findings, although some variability exists in the literature [27].

### 4.3. The “Topical Illusion” and Hidden Blood Loss

The term “Topical Illusion” refers to the apparent reduction in visible postoperative blood loss after intra-articular TXA administration, which may create an overestimation of overall blood conservation when hidden blood loss is not fully considered. A key finding of this study is the difference in observed efficacy between intravenous (IV) and intra-articular (IA) TXA administration. While IA TXA administration has gained popularity due to its perceived safety, our results indicate that it is associated with a limited effect on measured blood loss parameters [27,28,29]. The IA group achieved a marked reduction in 24 h drain output (*p* < 0.001), yet it failed to significantly impact TBL or HBL. This suggests that topical application may predominantly reduce visible postoperative drainage while having limited effect on calculated hidden blood loss components. Our data show that the IA TXA group achieved approximately 3.7% reduction in TBL and 8.2% in HBL compared to controls, whereas IV administration achieved a 30.3% reduction in TBL.

### 4.4. Therapeutic Ceiling Effect and Clinical Implications

The lack of a statistically significant difference in TBL between the IV TXA group (898 mL) and the combined IV + IA TXA group (896 mL) is an observation of interest within the limits of this study. This suggests a potential plateau effect of systemic TXA dosing under the present protocol. The findings presented in Figure 4 further support these observations by illustrating the percentage reduction in total and hidden blood loss across treatment groups. These results are consistent with the primary analyses and reinforce the absence of a statistically significant incremental benefit of combined IV and intra-articular administration over IV TXA alone. While some studies and meta-analyses suggest the superiority of combined protocols, our findings are broadly consistent with studies reporting no additional benefit of combined administration over IV TXA alone [29,30,31,32,33]. Alshryda et al. also reported significant reductions in blood loss and hospital stay with IV TXA [19]. Furthermore, although transfusion rates did not reach statistical significance, the absolute values showed a tendency in favor of systemic protocols without reaching statistical significance.

### 4.5. Clinical Validation Through Transfusion Correlation

The clinical importance of our volumetric calculations is supported by the distinct blood loss profiles between transfused and non-transfused patients. The overall transfusion rate of 6.9% is consistent with previous reports [32,33,34]. Transfused patients exhibited a median HBL nearly twice as high as non-transfused counterparts (1036 mL vs. 576.5 mL, *p* < 0.001), suggesting an association between higher calculated hidden blood loss and the likelihood of transfusion within this cohort. Notably, HBL accounted for approximately 72% of total blood loss (1036/1431 mL) in the transfused group. This suggests that hidden blood loss represents a substantial proportion of total blood loss. These findings suggest that both visible and hidden components of blood loss should be considered in perioperative management strategies [35].

### 4.6. Independent Predictors of Blood Loss

The multivariate linear regression model (Figure 6) provided insight into factors independently associated with total blood loss. After adjusting for potential confounders-including age, gender, BMI, and surgical duration, only the intravenous (IV) and combined protocols were found to be independent predictors of lower TBL. The IV route demonstrated a statistically significant association (B = −383.7, *p* = 0.001), indicating a reduction in total blood loss within the adjusted model. Conversely, intra-articular (IA) administration failed to reach statistical significance as an independent predictor, suggesting that IA administration alone was not significantly associated with reduced total blood loss in this analysis. Similar findings were reported in studies by Prakash et al. and Bi et al., supporting systemic or combined efficacy [36,37].

### 4.7. Preoperative Hemoglobin Paradox

An interesting finding was the observed association between higher preoperative Hb levels and higher calculated TBL. This is likely a mathematical reflection of the Gross formula, where a higher initial Hb concentration results in a larger calculated volume for the same Hb drop. Clinically, this suggests that higher preoperative Hb values do not eliminate the potential for clinically relevant perioperative blood loss, as absolute blood loss in these patients can still be substantial.

### 4.8. Limitations

This study has several limitations. First, although prospective, it was a single-center observational cohort study rather than a randomized trial. Second, transfusion data was limited by the relatively low number of events, which precluded a powered statistical comparison for this secondary outcome. Third, clinical datasets inherently contain potential outliers in laboratory parameters. Despite these limitations, the consistency across multiple outcome measures-calculated blood loss, hemoglobin changes, and multivariate analyses suggests internal consistency of the observed results.

## 5. Conclusions

Systemic administration of tranexamic acid (TXA), whether intravenous or combined, is associated with greater reductions in total and hidden blood loss compared with isolated intra-articular application following total knee arthroplasty. While intra-articular TXA effectively reduces visible 24 h drainage, it has a limited effect on calculated hidden blood loss and the subsequent decline in hematocrit levels. Furthermore, the addition of a local dose to a systemic IV protocol did not demonstrate an additional statistically significant benefit under the present study conditions, which may suggest a potential plateau effect of systemic TXA dosing. Therefore, a standalone intravenous TXA protocol may represent a viable option for perioperative blood conservation in TKA.

## Figures and Tables

**Table 1:** Baseline demographic and perioperative data across groups.

	Total (*n* = 245)	Control (*n* = 62)	TXA Intravenous (*n* = 61)	TXA Intra-Articular (*n* = 61)	TXA Combined (*n* = 61)	*p*
Gender						0.575 ^a^
Female	146 (59.6)	33 (53.2)	40 (65.6)	37 (60.7)	36 (59.0)	
Male	99 (40.4)	29 (46.8)	21 (34.4)	24 (39.3)	25 (41.0)	
Age	71 (8.5)	71.5 (7.5)	71 (6.9)	71 (9.5)	71 (8.5)	0.965 ^b^
Body height	170 (8.0)	171 (7.25)	170 (6.0)	170 (14.0)	170 (6.5)	0.816 ^b^
Body weight	86 (17.0)	87 (12.2)	86 (16.0)	86 (25.0)	85 (19.0)	0.266 ^b^
BMI	29.4 (4.2)	29.7 (2.9)	29.0 (3.5)	29.7 (5.9)	28.9 (4.7)	0.230 ^b^
Knee						0.370 ^a^
Left	99 (40.4)	23 (37.1)	30 (49.2)	25 (41.0)	21 (34.4)	
Right	146 (59.6)	39 (62.9)	31 (50.8)	36 (59.0)	40 (65.6)	
Duration of surgery (min)	120 (25.0)	120 (36.2)	120 (25.0)	120 (25.0)	120 (32.5)	0.969 ^b^

^a^ Pearson χ^2^ test; ^b^ Kruskal–Wallis test; Categorical variables are presented as *n* (%), and numeric as median (IQR).

**Table 2:** Laboratory parameters of patients in relation to the method of TXA administration.

	Total (*n* = 245)	Control (*n* = 62)	TXA Intravenous (*n* = 61)	TXA Intra-Articular (*n* = 61)	TXA Combined (*n* = 61)	*p* ^a^
Preoperative Hb	134 (21.0)	135 (23.0)	132 (21.5)	135 (19.0)	134 (21.5)	0.425
Postoperative Hb	106 (24.0)	105 (24.5)	110 (25.0)	104 (21.0)	109 (23.5)	0.228
Delta Hb	24 (18.0)	28 (21.0)	22 (11.5)	25 (23.5)	23 (16.0)	0.031
Preoperative HCT	0.397 (0.060)	0.410 (0.074)	0.394 (0.055)	0.396 (0.052)	0.392 (0.064)	0.217
Postoperative HCT	0.315 (0.065)	0.310 (0.066)	0.323 (0.057)	0.303 (0.061)	0.324 (0.068)	0.326
Delta HCT	0.073 (0.05)	0.079 (0.07)	0.069 (0.05)	0.079 (0.06)	0.065 (0.04)	0.009

^a^ Kruskal–Wallis test; Numerical variables are presented as median (IQR).

**Table 3:** Parameters of blood loss and drainage in relation to the method of TXA administration.

	Total (*n* = 245)	Control (*n* = 62)	TXA Intravenous (*n* = 61)	TXA Intra-Articular (*n* = 61)	TXA Combined (*n* = 61)	*p*
Transfusion *n* (%)	17 (6.9)	8 (12.9)	5 (8.2)	2 (3.3)	2 (3.3)	0.128 ^a^
EBV (mL)	4908 (1139.0)	4956.5 (953.0)	4750 (976.0)	4982 (1473.5)	4918 (1218.0)	0.422 ^b^
TBL (mL)	1014 (788.0)	1172 (1128.3)	898 (602.5)	1129 (935.5)	896 (623.5)	0.004 ^b^
HBL (mL)	635 (735.5)	814.5 (1095.3)	568 (655.0)	748 (894.0)	498 (767.0)	0.020 ^b^
Total aspiration and drainage (mL)	330 (230.0)	390 (240.0)	300 (250.0)	300 (240.0)	300 (280.0)	0.012 ^b^
Drain blood volume (mL)	180 (200.0)	260 (155.0)	100 (185.0)	150 (140.0)	150 (200.0)	<0.001 ^b^
Intraoperative blood loss (mL)	100 (120.0)	100 (77.5)	100 (127.5)	150 (145.0)	100 (195.0)	0.392 ^b^

^a^ Fisher exact test; ^b^ Kruskal–Wallis test; Categorical variables are presented as *n* (%), and numeric as median (IQR). c Post hoc analysis (Bonferroni) *p* = 0.004b (IV vs. IA: *p* = 0.009c; IV vs. Control: *p* = 0.002c); *p* = 0.02b (IV vs. IA: *p* = 0.008c; IV vs. Control: *p* = 0.019c; Combined IV + IA vs. IA: *p* = 0.036c).

**Table 4:** Multivariate linear regression analysis of factors associated with total blood loss (TBL).

Variable	B	95% CI for B	*p*
TXA intra-articular vs. control	−94.996	−314.359 to 124.367	0.394
TXA intravenous vs. control	−383.760	−605.099 to −162.422	0.001
TXA combined vs. control	−327.800	−547.907 to −107.693	0.004
Age	5.122	−6.464 to 16.708	0.385
Gender	47.149	−134.306 to 228.604	0.609
Duration of the operation	2.787	−0.399 to 5.974	0.086
Hb preoperatively	9.588	3.994 to 15.183	0.001
Platelets preoperatively	−0.401	−1.658 to 0.855	0.530
BMI	−7.197	−31.619 to 17.225	0.562

## Data Availability

The data presented in this study are not publicly available due to patient privacy and ethical restrictions. The data are available from the corresponding author upon reasonable request.

## Abbreviations

The following abbreviations are used in this manuscript:

TXA	Tranexamic acid
IV	Intravenous
IA	Intra-articular
TBL	Total blood loss
HBL	Hidden blood loss
TKA	Total knee arthroplasty
OA	Knee osteoarthritis
VTE	Venous thromboembolism
EBV	Estimated blood volume
Hg	Hemoglobin
